# Risk factors for early postoperative complications after minimally invasive surgery in pediatric ulcerative colitis

**DOI:** 10.1002/jpn3.70364

**Published:** 2026-02-02

**Authors:** Martina Di Benedetto, Luca Scarallo, Sara Renzo, Sara Naldini, Monica Paci, Jacopo Barp, Federico Perna, Emilio Paolo Emma, Riccardo Coletta, Stefano Scaringi, Paolo Lionetti

**Affiliations:** ^1^ Gastroenterology and Nutrition Unit Meyer Children's Hospital IRCCS Florence Italy; ^2^ Department of NEUROFARBA University of Florence Florence Italy; ^3^ Inflammatory Bowel Disease Surgical Unit Careggi University Hospital Florence Italy; ^4^ Surgical Unit Meyer Children's Hospital IRCCS Florence Italy

**Keywords:** inflammatory bowel diseases, laparoscopic approach, postsurgical outcomes, robotic approach

## Abstract

**Objective:**

To report a single‐center experience with a multidisciplinary minimally invasive surgical approach for pediatric ulcerative colitis (UC) and identify risk factors for early postoperative complications (EPC).

**Methods:**

A retrospective analysis was conducted on UC patients followed at the Gastroenterology Unit of Meyer Children's Hospital, who underwent surgery between 2010 and 2023.

**Results:**

Seventy‐four surgical procedures in 31 patients were analyzed. All patients underwent subtotal colectomy; 24 proceeded to ileal‐pouch‐anal anastomosis (IPAA), and 19 had completed ileostomy closure at the time of analysis. Twenty‐five (80.7%) colectomies were laparoscopic, and 6 (19.3%) were open. Among IPAA procedures, 20.8% (*n* = 5) were open, 50% (*n* = 12) were laparoscopic, and 29.2% (*n* = 7) were robotic. Eight patients (25.8%) experienced EPC after colectomy. Univariate analysis identified diagnosis before 6 years of age (very early onset inflammatory bowel disease) as a significant risk factor for EPC (*p* = 0.026; OR: 10.5; 95% CI: 1.4–38). Open and laparoscopic approaches showed comparable EPC rates (colectomy: 16.7% vs. 28%, *p* = 0.998; IPAA: 20% vs. 8.3%, *p* = 0.515). Laparoscopic surgery was associated with a significantly lower time to enteral feeding, bowel function recovery, and hospital discharge for both colectomies (*p* = 0.005, 0.002, and 0.025, respectively) and IPAA procedures (*p* = 0.008, 0.001, and 0.044, respectively). Robotic approach further shortened return of bowel function compared to both laparoscopic and to open approach (*p* = 0.032 and *p* = 0.002, respectively).

**Conclusions:**

Minimally invasive surgery for pediatric UC is safe and associated with improved postoperative recovery. Younger age and poor nutritional status may increase the risk of early complications. The robotic approach also shows promise in further improving recovery times.

## Abstract (highlights)

**What is Known:**

- The incidence of ulcerative colitis (UC) has been increasingly shifting toward younger patients, often presenting with more extensive and severe disease.
- Despite advances in biological therapies, 20%–30% of patients will ultimately require major surgery during their lifetime.
- The latest European Society for Pediatric Gastroenterology, Hepatology, and Nutrition (ESPGHAN) guidelines recommend a minimally invasive laparoscopic approach with a three‐stage surgical strategy for pediatric patients with UC.

**What is New:**

- The laparoscopic approach is safe and associated with improved recovery times compared to open surgery in pediatric UC, particularly when performed by an experienced multidisciplinary team.
- The robotic‐assisted technique for ileal pouch‐anal anastomosis is further associated with enhanced postoperative recovery.

## 1 INTRODUCTION

Over the past decade, the incidence of ulcerative colitis (UC) has increasingly shifted toward younger age.^1^, ^2^ While the clinical presentation of UC in children and adults is generally similar, specific differences have been reported. Pediatric‐onset UC tends to present with more extensive disease involvement.^3^, ^4^ Particularly, in pediatric UC, more than 75% of patients present with extended colitis or pancolitis, in contrast to adults, who primarily present with left‐sided disease.^5^, ^6^, ^7^, ^8^ Additionally, even when diagnosed as left‐sided disease, about 50% of children may progress to develop extensive colitis over time during follow‐up.^5^, ^9^ This progression may require escalation to biologic treatments, and in some cases, surgery is needed due to medical‐refractory disease.^10^, ^11^ Indeed, despite the advent of biological therapies, between 20% and 30% of patients will eventually require major surgery during their lifetime, with more than half of these patients needing surgical intervention within the first 5 years from diagnosis.^12^, ^13^

According to the last European Society for Pediatric Gastroenterology, Hepatology, and Nutrition (ESPGHAN) pediatric guidelines, surgery is recommended for children with acute severe colitis (ASC) refractory to medical therapy and in steroid‐dependent UC despite optimized medical treatment.^14^, ^15^ Surgical treatment for pediatric UC typically involves three stages: an initial subtotal colectomy with end‐ileostomy, followed by a restorative proctocolectomy with ileal‐pouch‐anal anastomosis (IPAA) or ileorectal anastomosis, and, finally, closure of the covering ileostomy.^14^ A minimally invasive laparoscopic approach is recommended for pediatric UC patients, offering outcomes comparable to open surgery in both urgent and elective settings.^14^ While numerous studies in adult populations indicate improved outcomes with laparoscopic procedures,^16^, ^17^, ^18^, ^19^, ^20^, ^21^, ^22^ evidence specifically addressing feasibility and both short‐ and long‐term outcomes of minimally invasive approaches in pediatric UC remains elusive.^23^, ^24^, ^25^, ^26^, ^27^

This study reports the experience of a single pediatric referral center managed by a multidisciplinary team of pediatric and adult colorectal surgeons. The primary objective was to identify potential risk factors for early postoperative complications (EPC) following surgical treatment of pediatric UC during a period of progressive adoption of minimally invasive approaches. Secondary outcomes focused on evaluating the potential advantages of minimally invasive techniques compared with the open approach.

## 2 METHODS

### 2.1 Ethics statement

Ethical approval was obtained by Meyer Children's Hospital IRCCS IRB (*approval protocol number: 180/2020*). Patient confidentiality was strictly maintained, and all data were anonymized for analysis. Informed consent was waived for this retrospective and anonymous investigation. The research was conducted according to Helsinki 2007 criteria.

### 2.2 Study population and data collection

We conducted a retrospective analysis of pediatric patients affected by UC followed up at the Pediatric Gastroenterology and Nutrition Unit of Meyer Children's Hospital IRCSS, who underwent abdominal surgery between January 1, 2010 and December 31, 2023.

All patients were diagnosed according to established clinical, endoscopic, and histological criteria.^14^, ^28^ Clinical, demographic, anthropometric, laboratory, and treatment data were retrieved from digital and nonelectronic charts at the time of diagnosis and surgery. For each patient, the following variables were collected: sex, age at diagnosis, presence of very early‐onset inflammatory bowel disease (VEO‐IBD), family history of inflammatory bowel disease (IBD), body mass index (BMI) at diagnosis, age and BMI at the time of surgery (according World Health Organization table for age and sex), duration of disease before surgery, and disease phenotype according to the Paris Classification and changes in disease extent over time. Disease activity was evaluated by the Pediatric Ulcerative Colitis Activity Index (PUCAI).^29^ Indication for colectomy—categorized as acute severe UC or failure of medical therapy—was also recorded.

### 2.3 Surgical setting and multidisciplinary team

Surgical procedures were performed by combined team: pediatric surgeons from the Pediatric Surgical Unit at Meyer Children's Hospital and adult colorectal surgeons from the IBD Unit at Careggi Hospital. All procedures were performed at Meyer Children's Hospital, within a dedicated pediatric setting that included pediatric gastroenterologists, anesthesiologists, dietitians, nurses, and specialized support staff. The choice of lead surgeon for each operation was primarily determined by the patient's age and the complexity of the procedure. In general, children under the age of 10 were operated on by the pediatric surgeon as the primary operator, whereas more complex procedures—such as IPAA—were led by an adult colorectal surgeon, in accordance with current guidelines recommending that pouch surgery be performed by experienced surgeons who carry out at least 10 pouch procedures annually.^15^ In all cases, a second surgeon from the collaborating team was present and actively involved, ensuring a dual‐specialist approach throughout the procedure.

After surgery, all patients were admitted to the Pediatric Surgery Unit or, in selected cases, to the Pediatric Intensive Care Unit of our hospital. Postoperative management followed a shared care pathway coordinated by a multidisciplinary team comprising pediatric gastroenterologists, dietitians, and surgeons. Since the early 2010s, postoperative care at our institution has been standardized through the implementation of an internal perioperative protocol routinely applied to pediatric patients undergoing intestinal surgery. This protocol promotes the early initiation of enteral feeding—ideally within 24 h postoperatively—when specific clinical criteria are met, including hemodynamic stability, minimal nasogastric output, absence of vomiting, and evidence of bowel function recovery.

### 2.4 Study outcomes

EPC were defined as any surgery‐related morbidity occurring within 30 days from the procedure and were classified into three categories^1^; intra‐abdominal infectious complications^2^; mechanical complications (intestinal obstruction); and^3^ other complications, such as wound dehiscence or bowel perforation. The severity of postoperative complications was assessed using the Clavien‐Dindo Classification. The latter includes five grades: (1) minor complications that do not require pharmacological treatment or surgical intervention; (2) complications requiring pharmacological treatment (e.g., antibiotics or blood transfusion); (3) complications requiring surgical, endoscopic, or radiological intervention, with IIIa involving local anesthesia or sedation, and IIIb requiring general anesthesia; (4) life‐threatening complications, with IVa involving single organ failure and IVb involving multi‐organ failure; and (5) death due to complications.^30^ Postoperative follow‐up for monitoring early complications was performed daily during the hospital stay, followed by outpatient visits at 2 and 4 weeks after discharge, ensuring a minimum follow‐up period of 30 days for all patients.

Data were analyzed to identify predictors of EPC. For each stage (subtotal colectomies and ileal pouch‐anal anastomosis), we compared the two groups (patients with and without early complications) across the following characteristics: age at diagnosis (years), diagnosis of VEO‐IBD, age at surgery (years), disease duration before surgery (months), BMI at surgery and BMI *Z*‐score at surgery, PUCAI score at surgery, surgical indication, medical treatments within 4 weeks before surgery (steroids, biologics, immunomodulators, thalidomide), need for emergency surgery, type of lead surgeon (pediatric or adult colorectal surgeon), length of postoperative hospital stay (days), time to enteral feeding (days), and time to return of bowel function (days).

Postsurgical short‐term outcomes included the number of EPC, time to enteral feeding (days), time to return of bowel function (days), and length of postoperative hospital stay (days). For subtotal colectomy, we compared open and laparoscopic approaches. For IPAA, we compared open and laparoscopic approaches, as well as laparoscopic and robotic approaches and open and robotic approaches.

### 2.5 Statistical analysis

Data were managed and analyzed using IBM SPSS Statistics, version 25.0 (IBM Corp.).^31^ Categorical variables were summarized as frequencies and percentages. The distribution of continuous variables was assessed using the Kolmogorov–Smirnov goodness‐of‐fit test, and none were found to follow a normal distribution. Consequently, nonnormally distributed continuous variables were reported as medians with interquartile ranges (IQRs). Statistical differences among categorical variables were evaluated using the *χ*² test or Fisher's exact test, when appropriate. Comparisons of nonnormally distributed continuous variables were performed using the Mann–Whitney *U*‐test or the Wilcoxon signed‐rank test. A *p*‐value of < 0.05 was considered statistically significant.

## 3 RESULTS

A total of 74 surgical procedures involving 31 patients with UC were analyzed. All 31 patients underwent subtotal colectomy as the initial surgical stage. Subsequently, IPAA was performed in 24 of the 31 patients as the second surgical step, followed by ileostomy closure in 19 patients as the third stage. At the time of data analysis, the remaining patients had either not yet progressed to the subsequent surgical stages or had been transferred to the adult center before completing the surgical program.

### 3.1 Characteristics of the population

Population characteristics and clinical data at diagnosis and at the first surgical stage are summarized in Table 1. According to the Paris Classification, disease localization at diagnosis was E2 in 12 patients (38.7%), E3 in 4 patients (12.9%), and E4 in 15 patients (48.4%). At the time of surgery, localization was E2 in 6 patients (19.4%), E3 in 6 patients (19.4%), and E4 in 19 patients (61.3%). Eight patients (25.8%) presented disease extension over time before colectomy. At diagnosis, 8 patients (25.8%) had ASC, while by the time of surgery, 17 patients (54.8%) had experienced at least one episode of ASC. The median PUCAI score was 35 (IQR: 30–60) at diagnosis and 50 (IQR: 45–65) at surgery. The indication for surgery was acute severe UC in 8 patients (25.8%) and failure of medical treatment in 23 patients (74.2%).

**Table 1:** Characteristics of the population.

Characteristics	Overall cohort (*n* = 31)	
**Sex (male)**	16 (51.6%)	
**Median age at diagnosis (years)**	10.8 (IQR: 7.6–14.1)	
**VEO‐IBD**	6 (19.4%)	
**Familiarity for IBD**	7 (22.6%)	
**Median BMI at diagnosis** (kg/m^2^)	18.6 (IQR: 16.7–21.9)	
**Median age at surgery** (years)	13.5 (IQR: 10.4–15.2)	
**Median BMI at surgery** (kg/m^2^)	17.4 (IQR: 16–4‐21.9)	
**Median duration of disease before surgery** (months)	16.8 (IQR: 5.2–42.9)	
**Paris classification**	**At diagnosis**	**At first surgery**
Localization	E2 12 (38.7%) E3 4 (12.9%) E4 15 (48.4%)	E2 6 (19.4%) E3 6 (19.4%) E4 19 (61.3%)
Characteristics	S0 23 patients (74.2%) S1 8 patients (25.8%)	S0 14 patients (45.2%) S1 17 patients (54.8%)
**Change in disease's extension**	8 (25.8%)	
**PUCAI**	**At diagnosis**	**At first surgery**
Median	35 (IQR: 30–60)	50 (IQR: 45–65)
Score	Mild (15–34): 8 (25.8%) Moderate (35–64): 16 (51.6%) Severe (> 65): 7 (22.6%)	Mild (15–34): 0 Moderate (35–64): 23 (74.2%) Severe (> 65): 8 (25.8%)
**Indication to colectomy** Acute severe ulcerative colitis Failure medical treatment	8 (25.8%) 23 (74.2%)	

Abbreviations: BMI, body mass index; IBD, inflammatory bowel disease; IQR, interquartile range; PUCAI, pediatric ulcerative colitis activity index; VEO‐IBD, very early onset inflammatory bowel disease.

### 3.2 Early postsurgical complications

#### 3.2.1 Subtotal colectomy

A total of 31 subtotal colectomies were analyzed; 8 (25.8%) experienced EPC. These included three infectious complications (classified as Grade II, IIIa, and IIIb according to the Clavien‐Dindo Classification), one of which was an abdominal abscess requiring percutaneous drainage; four cases of intestinal subobstruction necessitating ileostomy revision surgery with adhesiolysis and stoma refashioning (Grade IIIb); and one duodenal perforation complicated by septic shock (Grade IV).

Six out of eight procedures complicated by EPC were performed by pediatric surgeons; five of these were performed laparoscopically, and four involved patients with very‐early onset IBD.

In the univariate analysis, the only factor significantly associated with a higher risk of postoperative complications was a diagnosis of VEO‐IBD (OR: 10.5; 95% CI: 1.4–78; *p* = 0.026). A multivariate analysis could not be performed due to the small sample size. A statistically significant difference in median BMI at the time of surgery was observed between patients who experienced postoperative complications and those who did not. This difference was evident both in absolute BMI (*p* = 0.009) and as a *Z*‐score BMI (*p* = 0.055).

None of the other demographic or clinical variables summarized in Table 2 was significantly associated with postoperative complications. Notably, none of the medical treatments administered 4 weeks before surgery was associated with a higher risk of early complications.

**Table 2:** Early postoperative complications after colectomy procedures.

	Colectomies (*n* = 31)		
	No early complications (*n* = 23, 74.2%)	Early complications (*n* = 8, 25.8%)	*p*‐Value
Median age at diagnosis (years)	11.5 (IQR: 8.3–14.4)	6 (IQR: 2.6–12.3)	0.095
VEO‐IBD	2 (8.7%)	4 (50%)	0.026*
Median age at surgery (years)	14.1 (IQR: 11–15.8)	10.1 (IQR: 5.3–15)	0.321
Median duration of disease before surgery (months)	15.5 (IQR: 3–40.4)	22.7 (IQR: 16.7–47.7)	0.125
Median BMI at surgery (kg/m^2^)	18.1 (IQR: 16.8–22.1)	16.3 (IQR: 15.9–16.3)	0.009*
Median BMI *Z*‐score at surgery	0 (IQR: −0.8 to 1.5)	−1.8 (IQR: −1.9 to −0.7)	0.055
Median PUCAI at surgery	50 (IQR: 45–65)	53 (IQR: 50–58)	0.766
Indication to surgery Acute severe colitis Failure medical treatment	7 (30.4%) 16 (69.6%)	1 (12.5%) 7 (87.5%)	0.642
Medical treatments 4 weeks before surgery			
Endovenous steroids	16 (69.6%)	7 (87.5%)	0.642
Biologics Infliximab Vedolizumab Ustekinumab	20 (87%) 14 (60.9%) 5 (21.7%) 1 (4.3%)	6 (75%) 3 (37.5%) 2 (25%) 1 (12.5%)	0.583 0.412 1 0.456
Immunomodulators Azatioprine Ciclosporine	6 (26.1%) 1 (4.3%) 5 (21.7%)	3 (37.5%) 2 (25%) 1 (12.5%)	0.660 0.156 1
Talidomide	2 (8.7%)	3 (37.5%)	0.093
Urgent surgery	10 (43.5%)	6 (75%)	0.220
Lead surgeon Pediatric Adult	9 (39.1%) 14 (60.9%)	6 (75%) 2 (25%)	0.090
Surgical approach Laparotomic Laparoscopic	5 (21.8%) 18 (78.2%)	1 (12.5%) 7 (87.5%)	1
Median length of postsurgical hospital stay (days)	8 (IQR: 7–13)	21 (IQR: 9–35)	0.053
Median time to enteral feeding (days)	2 (IQR: 1–3)	2 (IQR: 1–7)	0.651
Median time to return of bowel function (days)	1 (IQR: 1–2)	1 (IQR: 1–3)	0.749

*Note*: Bold values indicate statistically significant (*p* < 0.05).

Abbreviations: BMI, body mass index; IQR, interquartile range; PUCAI, pediatric ulcerative colitis activity index; VEO‐IBD, very early‐onset inflammatory bowel disease.

#### 3.2.2 Ileo‐pouch‐anal anastomosis

Among the 24 ileo‐pouch‐anal anastomosis surgeries, 3 patients experienced EPC. These included 1 intra‐abdominal infectious event (IIIa according to the Clavien‐Dindo classification) and two instances of small bowel obstructions (IIIb according to the Clavien‐Dindo classification). None of the demographic or clinical variables analyzed were significantly associated with the occurrence of postoperative complications, as summarized in Table S1.

#### 3.2.3 Ileostomy closure

One early complication was observed, specifically a mechanical complication (stoma outlet obstruction) classified as grade 3b according to the Clavien‐Dindo classification.

### 3.3 Open versus minimally invasive approach

#### 3.3.1 Subtotal colectomy

Of the 31 subtotal colectomies analyzed, 6/31 (19.3%) were performed using a laparotomic (open) approach and 25/31 (80.7%) using a laparoscopic approach. All colectomies done via laparotomy were carried out in the early 2010s, suggesting a gradual shift toward minimally invasive techniques in more recent years. No statistically significant differences were observed between the two groups in terms of the clinical characteristics analyzed, as reported in Table 3.

**Table 3:** Comparison between open and laparoscopic approaches in colectomy procedures.

	Colectomies (*n* = 31)		
	Open (*n* = 6, 19.3%)	Laparoscopic (*n* = 25, 80.7%)	*p*‐Value
**Characteristics**			
Median age at surgery (years)	14.4 (IQR: 13.6–14.9)	13.1 (IQR: 10.4–15.2)	0.549
Median PUCAI at surgery	50 (IQR: 45–55)	55 (IQR: 45–65)	0.404
Median BMI at surgery (kg/m^2^)	17.4 (IQR: 16.9–20.1)	17.3 (IQR: 16.3–21.9)	0.685
Median duration of disease before surgery (months)	8 (IQR: 0–20.9)	17.7 (IQR: 10.9–42.9)	0.147
Indication to surgery Acute severe colitis Failure medical treatment	0 6 (100%)	8 (32%) 17 (68%)	0.298
Urgent surgery	5 (83.3%)	11 (44%)	0.172
Lead surgeon Pediatric Adult	4 (66.7%) 2 (33.3%)	11 (44%) 14 (56%)	0.394
**Postsurgical outcomes**			
Early postoperative complications	1 (16.7%)	7 (28%)	0.998
Median time to enteral feeding (days)	7 (IQR: 5–7)	2 (IQR: 1–3)	0.005*
Median time to return to bowel function (days)	3 (IQR: 3–4)	1 (IQR: 1–1)	0.002*
Median length of postsurgical hospital stays (days)	23 (IQR: 14–31)	9 (IQR: 7–12)	0.025*

*Note*: Bold values indicate statistically significant (*p* < 0.05).

Abbreviations: BMI, body mass index; IQR, interquartile range; PUCAI, pediatric ulcerative colitis activity index.

In terms of postsurgical outcomes, laparoscopic group showed significantly shorter median times to enteral feeding, to return to bowel function, and postoperative hospital stay compared to laparotomic approach (*p* = 0.005, 0.002, and 0.025, respectively).

#### 3.3.2 IPAA

A total of 24 IPAA were analyzed, with 5 (20.8%) performed using a laparotomic approach, 12 (50%) with a laparoscopic‐assisted approach, and 7 (29.2%) using a robotic approach. Data are summarized in Table 4.

**Table 4:** Comparison between open and laparoscopic and robotic approaches in IPAA procedures.

	IPAA (*n* = 24)								
	Open (*n* = 5; 20.8%)	Laparoscopic (*n* = 12; 50%)	*p*‐Value	Laparoscopic (*n* = 12; 50%)	Robotic (*n* = 7; 29.2%)	*p*‐Value	Open (*n* = 5; 20.8%)	Robotic (*n* = 7; 29.2%)	*p*‐Value
**Characteristics**									
Median time between first stage and second stage (months)	14.9 (IQR: 8.1–6.1)	10.4 (IQR: 6.2–6.5)	0.673	10.4 (IQR: 6.2–6.5)	9 (IQR: 6.1–6.4)	0.800	14.9 (IQR: 8.1–6.1)	9 (IQR: 6.1–6.4)	0.570
Lead surgeon Pediatric Adult	3 (60%) 2 (40%)	1 (8.3%) 11 (91.7%)	0.053	1 (8.3%) 11 (91.7%)	1 (14.3%) 6 (85.7%)	1	3 (60%) 2 (40%)	1 (14.3%) 6 (85.7%)	0.222
**Postsurgical outcomes**									
Early postoperative complications	1 (20%)	1 (8.3%)	0.515	1 (8.3%)	1 (14.3%)	1	1 (20%)	1 (14.3%)	1
Median time to enteral feeding (days)	5 (IQR: 5–7)	2 (IQR: 2–3)	0.008*	2 (IQR: 2–3)	2 (IQR: 1–2)	0.079	5 (IQR: 5–7)	2 (IQR: 1–2)	0.003*
Median time to return of bowel function (days)	3 (IQR: 3–3)	2 (IQR: 1–2)	0.001*	2 (IQR: 1–2)	1 (IQR: 1–1)	0.032*	3 (IQR: 3–3)	1 (IQR:1–1)	0.002*
Median length of postsurgical hospital stay (days)	15 (IQR: 10–16)	7 (IQR: 6–12)	0.044*	7 (IQR: 6–12)	7 (IQR: 5–10)	0.669	15 (IQR: 10–16)	7 (IQR: 5–10)	0.018*

*Note*: Bold values indicate statistically significant (*p* < 0.05).

Abbreviations: IPAA, ileal‐pouch‐anal anastomosis; IQR, interquartile range.

A trend was observed indicating a technical preference for the open approach among pediatric surgeons when comparing open versus laparoscopic procedures (*p* = 0.053). No significant differences were observed in EPC (*p* = 0.515). However, the laparoscopic group had a shorter median time to enteral feeding, return of bowel function, and postoperative hospital stay (*p* = 0.008, *p* = 0.001, and *p* = 0.044, respectively). When comparing the laparoscopic and robotic approaches, no statistically significant differences were found for the variables analyzed, except for a shorter median time to return of bowel function in the robotic group (*p* = 0.032). When comparing the open and robotic approaches, the robotic approach demonstrated a shorter median time to enteral feeding, return of bowel function, and postoperative hospital stay (*p* = 0.003, *p* = 0.002, and *p* = 0.018, respectively).

#### 3.3.3 Ileostomy closure

Nineteen patients underwent the third surgical stage of ileostomy closure, with 10 procedures (52.6%) performed by a pediatric surgeon, and 9 (47.4%) of 19 were conducted by an adult colorectal surgeon.

## 4 DISCUSSION

In this study, we have presented data from a sizeable cohort of pediatric UC patients who underwent surgery in the biologic era, managed by a multidisciplinary surgical team comprising both pediatric and adult colorectal surgeons, in the context of an increasing adoption of minimally invasive techniques over the past decade in the surgical management of pediatric UC. Our findings demonstrate that the laparoscopic approach is safe, not associated with a higher rate of postoperative complications, and linked to improved recovery times compared to open surgery. A younger age at the time of the first surgical procedure and poorer nutritional status emerged as potential risk factors for EPC. Additionally, we report a case series of children undergoing IPAA using a robotic‐assisted technique, which was further associated with reduced recovery times.

Current pediatric guidelines recommend a laparoscopic approach as the preferred surgical method for UC surgery in children, emphasizing that experienced high‐volume surgical teams should perform such complex procedures.^14^, ^15^ Furthermore, it is recommended that pouch surgery for children with UC should be performed by experienced pediatric or adult surgeons performing at least 10 pouches per year. However, such recommendations often conflict with the realities of pediatric surgical centers, where pediatric surgeons may lack a high‐volume number of patients. To address such a significant issue, at our center, since the early 2010s, we established a multidisciplinary team comprising both pediatric and experienced adult colon‐rectal surgeons working in a high‐volume center. This collaboration aims to reverse the trend of pediatric hospitals of referring patients to adult hospitals for surgery due to the limited experience specially for pouch surgery. In addition, such collaboration offers the opportunity to the young patients to undergo surgery in a pediatric setting.

Within our multidisciplinary team, in terms of lead surgeon, we observed no significant differences between pediatric and adult surgeons regarding the choice of technique for subtotal colectomy procedures. However, for ileal pouch‐anal anastomosis, adult colorectal surgeons were more likely to use a laparoscopic approach compared to their pediatric counterparts. This trend likely reflects differences in prior exposure and training in minimally invasive techniques. Importantly, no statistically significant difference was observed in postoperative complication rates between the two types of lead surgeons (*p* = 0.090), likely due to small sample size. However, it is notable that six out of the eight complications following colectomy occurred in procedures performed by pediatric surgeons, with five of these six cases conducted using a laparoscopic approach. These findings emphasize the crucial role of multidisciplinary collaboration—particularly between pediatric and adult colorectal surgeons—in optimizing surgical planning and decision‐making for complex pediatric IBD cases. They also reinforce the importance of maintaining adequate annual procedural volumes, especially for advanced laparoscopic techniques, as recommended by current guidelines to ensure safety and improve outcomes.^15^

Regarding EPC, a diagnosis of VEO‐IBD emerged as a potential risk factor for early complications following colectomy. However, due to the small sample size, we were unable to perform a multivariate analysis, so this result may be influenced by other variables. In the literature, although limited studies have explored this association, a multicenter study by the Porto Group of ESPGHAN indicated that children under 10 years of age at the time of colectomy are at an increased risk of postoperative complications.^32^ In our cohort, while the difference was not statistically significant, patients who developed complications had a lower median age at the time of surgery. This finding further underscores the need for heightened clinical and surgical vigilance in the perioperative management of younger patients. Additionally, a lower BMI at the time of surgery was significantly associated with a higher risk of complications. These results align with previous studies and current guidelines, which recommend optimizing nutritional status before surgery when feasible to reduce the risk of surgical complications.^14^, ^15^, ^33^ However, it should be considered that frequently in patient with UC who undergo urgent colectomy, there is no time to improve nutritional status. In such cases, a three‐stage procedure with colectomy and ileostomy at first is recommended.

Regarding medications administered in the 4 weeks before subcolectomy, none were associated with an increased risk of postsurgical complications. The latest pediatric guidelines recommend suspending anti‐tumor necrosis factor‐α (TNF‐α) therapy 4–6 weeks before surgery.^14^ The European Crohn's and Colitis Organisation (ECCO) 2022 guidelines on UC surgical management in adults suggested a potential increase in early or late pouch‐specific complications with biologics, although no specific suspension timing was provided.^34^ However, the updated 2024 ECCO guidelines on the surgical management of Crohn's disease advise against suspending biologic therapies before surgery, citing a lack of evidence for increased postsurgical complications in patients treated with anti‐TNF agents, Vedolizumab, or Ustekinumab.^35^

Our findings indicate that the laparoscopic approach is both feasible and safe, with no statistically significant differences in EPC when compared to open surgery, in line with previously reported evidence.^23^, ^36^ In our cohort, the primary advantage of the laparoscopic‐assisted approach for both subcolectomy and IPAA surgeries was a reduction in postsurgical enteral feeding and return to bowel function times, as well as a shorter postoperative hospital stay. These results align with several meta‐analyses and clinical studies in adult populations, which have consistently shown that laparoscopic surgery is associated with reduced postoperative pain, faster bowel function recovery, and shorter hospitalization, without compromising safety outcomes.^16^, ^17^, ^18^, ^19^, ^20^, ^21^, ^22^ Although pediatric data remain limited, a recent meta‐analysis confirms comparable benefits in children—particularly shorter hospital stays and fewer postoperative complications—highlighting the importance of tailoring the surgical approach to both the surgeon's expertise and the individual patient's characteristics.^26^

In our experience, seven children underwent IPAA surgery with a robotic approach. Currently, no large‐sample studies in the literature compare the laparoscopic and robotic approaches for UC surgery in pediatric patients, though adult studies indicate a strong safety profile for the robotic approach.^37^, ^38^, ^39^ The first case series, published in 2011,^40^ demonstrated the feasibility of robotic proctocolectomy with J‐pouch creation. Early case‐matched studies comparing robotic and laparoscopic proctectomy with IPAA for IBD indicate similar effectiveness across measures such as hospital stay, return of bowel function, postoperative complications, and short‐term pouch function.^41^, ^42^ Although the number of robotic cases was limited (*n* = 7), our experience suggests that the robotic and laparoscopic approaches are comparable in terms of safety and postoperative hospital stay. The robotic approach showed a trend toward shorter refeeding times and a significantly faster return of bowel function. These findings should be interpreted with caution and considered preliminary, highlighting the need for further studies with larger sample sizes.

This study has several limitations. First, its retrospective design inherently introduces potential sources of bias, such as selection bias and incomplete data collection. Second, the relatively small sample size did not allow for multivariate analysis, limiting the statistical power, the robustness of the conclusions, and the generalizability of the findings to broader populations or different clinical settings. Postoperative pain and cosmetic outcomes were also not objectively measured, despite the well‐established cosmetic benefits of minimally invasive surgery.

## 5 CONCLUSION

In this single‐center experience, the progressive implementation of minimally invasive techniques within a multidisciplinary pediatric–adult surgical model proved to be safe and effective for the management of pediatric UC. Minimally invasive approaches, when performed by an experienced surgical team, were not associated with an increased risk of EPC and were instead linked to faster postoperative recovery. Younger age at the time of surgery and poorer nutritional status emerged as potential risk factors for postoperative complications, underscoring the importance of careful perioperative assessment and optimization. Robotic‐assisted IPAA showed promising early outcomes, although further studies with larger cohorts are necessary to confirm its advantages.

## CONFLICT OF INTEREST STATEMENT

The authors declare no conflicts of interest.

## Supporting information

Supplemental Table1‐clean.
