# Supplementary material for: Risk factors for early postoperative complications after minimally invasive surgery in pediatric ulcerative colitis
Source: J Pediatr Gastroenterol Nutr. 2026 Feb 2;82(4):1040–50. doi: 10.1002/jpn3.70364 (PMC13050802; doi:10.1002/jpn3.70364)
Supplement: Supplementary file 1 — Supplemental Table1‐clean. [file JPN3-82-1040-s001.docx]

|  | **Ileal pouch-anal anastomosis (n=24)** | |  |
| --- | --- | --- | --- |
|  | **No early complications**  **(n=21, 87.5%)** | **Early complications**  **(n=3, 12.5%)** | **p-value** |
| Median time between 1^st^ stage and 2^nd^ stage (months) | 10.2 (IQR:6.4-15.1) | 17.8 (IQR:2-19.5) | 0.570 |
| Median BMI at surgery (kg/m2) | 19 (IQR:17.3-24.8) | 18.1 (IQR:17.3-18.9) | 0.472 |
| Lead surgeon  Pediatric  Adult | 3 (14.3%)  18 (85.7%) | 2 (66.7%)  1 (33.3%) | 0.099 |
| Median length of post-surgical hospital stay (days) | 8 (IQR:7-10) | 24 (IQR:3-28) | 0.401 |
| Median time to enteral refeeding (days) | 2 (IQR:2-3) | 7 (IQR:2-20) | 0.076 |
| Median time to return of bowel function (days) | 2 (IQR:1-2) | 2 (IQR:1-4) | 0.513 |

**Supplemental Table 1.** Early post-operative complications after IPAA procedures.

Abbreviations: BMI – Body Mass Index. IQR – Interquartile Range.
